# Importance of quadratus lumborum muscle trigger point injection and prolotherapy technique for lower back and buttock pain

**DOI:** 10.3389/fpain.2022.997645

**Published:** 2022-11-22

**Authors:** Soo-Ji Sirh, So-Woon Sirh, Hah-Yong Mun, Heon-Man Sirh

**Affiliations:** ^1^ Department of Neurosurgery, Sirh's Private Pain Clinic, Seoul, South Korea; ^2^ Department of Anesthesiology and Pain Medicine, Wiltse Memorial Hospital, Suwon-si, South Korea; ^3^ Department of Neurosurgery, Yangju Armed Forces Hospital, Yangju-si, South Korea; ^4^ Department of Anesthesiology and Pain Medicine, Sirh's Private Pain Clinic, Seoul, South Korea

**Keywords:** quadratus lumborum muscle enthesopathy, quadratus lumborum trigger point, trigger point injection, integrated injection technique, quadratus lumborum myofascial pain

## Abstract

**Background:**

Low back pain is a heterogeneous disease. Myofascial pain and enthesopathy of the quadratus lumborum muscle are important causes of lower back and/or buttock pain. However, a concrete, safe, and effective injection technique for the treatment of trigger points and enthesopathy in the quadratus lumborum muscle has not yet been developed.

**Objectives:**

We aimed to evaluate the importance of the quadratus lumborum muscle and introduce an effective landmark-based blind injection technique for treating quadratus lumborum trigger points and enthesopathy.

**Methods:**

Adult patients (*n* = 17) with lower back and/or buttock pain were placed in the lateral decubitus position. Next, we delicately palpated the quadratus lumborum muscle to accurately locate its lesions, including trigger points, taut bands, and tendon lesions, after five key landmarks had been identified. A newly designed 60–90-mm, 28G thin hypodermic needle was inserted at the tender points. The needle was typically advanced until its tip touched the transverse process to treat myofascial trigger points and tendon lesions in the iliolumbar and lumbocostal fibers, excluding superficial trigger points of the iliocostal fibers. Subsequently, lidocaine (0.5%) or a mixture of lidocaine (0.5%) and dextrose (12.5–15%) was injected.

**Results:**

The pretreatment visual analog scale score for all 17 patients decreased from ≥4–8/10 (mean 5.588) to 0–1/10 (mean 0.294) after completion of all treatments. The total number of treatments was one to four in acute and subacute cases and two to eight in chronic cases. The mean follow-up period was 73.5 days (treatment period: range, 4 to 43 days + at least 60 days of follow-up).

**Conclusions:**

Herein, we describe for the first time a landmark-based integrated injection technique for the treatment of trigger points and myofascial pain in the quadratus lumborum; this technique is safe, effective, and can be used with or without steroids, fluoroscopy, or ultrasound guidance.

## Introduction

Low back pain is one of the most common diseases responsible for years lived with disability (1), and there is a 50% to 85% chance of developing low back pain during one's lifetime; in 10% to 23% of cases, the pain becomes chronic (2–4). Low back pain is a heterogeneous disease and is of unknown etiology in some chronic patients (5, 6). There are various causes, including lumbar herniated nucleus pulposus, stenosis, spondylosis, ligament and tendon lesions, and myofascial etiologies, including trigger points (TrPs), enthesopathy, partial tear, and fibrosis. Identifying the underlying cause associated with the symptoms on radiological examinations or ultrasonography is difficult. Many pain physicians consider low back pain and disc herniation or spinal stenosis with resultant radiculopathy to be frequently concurrent. In many cases, radiological findings, including magnetic resonance imaging (MRI), do not correlate with clinical symptoms (7).

Myofascial pain syndrome of the quadratus lumborum muscle is one of the most common musculoskeletal disorders among patients with low back and buttock pain, including failed back surgery syndrome (8). However, the functions and biomechanics of the quadratus lumborum muscle are currently obscure (9), despite the precise description by Travell & Simons (10). Therefore, the importance and proper injection technique for quadratus lumborum TrPs and enthesopathy have been frequently overlooked and underestimated. The quadratus lumborum muscle comprises several fascicles: iliocostal, iliolumbar, iliothoracic (less common than other fascicles), and lumbocostal (9, 10). The referral pain of the deep fiber TrPs extends from the lower back to the sacroiliac joint and subsequently to the lower buttock, while the referral pain of the superficial fiber TrPs could extend from the iliac crest to the greater trochanter, the lateral aspect of the femur, and the groin area (10). Given this physiology, quadratus lumborum myofascial pain can be responsible for pseudo-sciatica and failed back surgery syndrome when accompanied by sacroiliac joint or gluteus minimus TrPs (8, 10, 11).

The recently reported that a quadratus lumborum plane block using ultrasound is primarily used to manage perioperative pain or provide local anesthesia (12). However, to the best of our knowledge, there are no reports in the literature that concretely describe trigger point injection (TPI) or any integrative injection technique combining TPI and prolotherapy to safely and effectively treat pain associated with quadratus lumborum muscle lesions, such as TrPs, fibrosis, calcifications, or tendon lesions, either blind (landmark-based) or using ultrasound or other types of guidance.

Therefore, conventional TPI or quadratus lumborum block would not be a fundamental treatment to safely and effectively treat myofascial pain syndrome in the quadratus lumborum, which may account for a large proportion of nonspecific low back pain. The injection therapy known as prolotherapy, which uses a dextrose solution as the injectant, has been reported to effectively treat chronic musculoskeletal pain, including low back pain (13). Considering the benefits of TPI and prolotherapy, both techniques could be used together (14).

Here, we introduce and describe a landmark-based quadratus lumborum muscle TPI and integrative injection technique combining TPI with prolotherapy to safely and effectively treat quadratus lumborum pain.

## Methods

Ethical approval for this retrospective chart review study was granted by the Institutional Review Board (IRB) of Wiltse Memorial Hospital Joint Research Ethics Committee in Suwon, Korea (2021-W11). The requirement for written informed consent was waived by the IRB owing to the retrospective study design. All procedures were performed in accordance with the Declaration of Helsinki.

### Patient selection

#### Inclusion criteria

We included 17 patients (Table 1) with a diagnosis of quadratus lumborum myofascial pain syndrome who were treated at Dr. Sirh's Pain Clinic between March 1, 2020, and March 31, 2021. All patients had lower back and/or buttock pain, with or without thigh pain and paresthesia, with visual analog scale scores ≥4/10, and did not respond to medical therapy, physical therapy, manual therapy, or other conservative treatments.

**Table 1:** Demographic data of patients, visual analog scale scores, and clinical findings.

Age (years)/sex, duration	VAS scores		Number of procedures	Total period of treatment (days)	Previous Tx history	Location, physical findings
	Before Tx	At the end of Tx				
54/F, 2 weeks, gradually	6	0	2	4	NSAIDs medication 2 weeks (ibuprofen)	Bilateral transverse back pain, pain aggravation with flexion ROM
42/M, 6 months, gradually	6	0	8	43	Lumbar steroid root block	Lt. buttock pain, pain aggravation with flexion ROM
40/M, 3 days, suddenly	4	0	4	8	Acupuncture	Lt. buttock pain, pain aggravation with extension, Rt. side bending (Lt. side pain) ROM
41/M, 3 months, after baby bathing	6	0	8	22	Medication (ibuprofen), acupuncture, physical therapy	transverse back pain, pain aggravation with flexion, extension ROM
41/F, 6 months, suddenly	6	0	5	20	Osteopathic Tx, medication (NSAIDs)	Bilateral back and buttock pain Difficult to stand, position change, pain aggravation with both side rotation ROM
62/M, 5 days, suddenly after truncal rotation	8	1	3	5	Medication (NSAIDs)	Rt. buttock pain Pain aggravation with position change (sitting->standing), flexion, extension, both side rotation LOM
40/M, over 2 years, gradually	6	1	3	7	Medication (acetaminophen), osteopathic Tx	Bilateral back pain Pain aggravation with flexion ROM
62/M, 1 week, suddenly	6	1	3	6	Medication (ibuprofen), acupuncture	Rt. side lumbar to buttock vertical pain Pain aggravation with extension ROM
30/M, 1 week, after climbing	6	0	2	7	Medication (ibuprofen)	Bilateral back pain with extension ROM
60/M, 2 days, after driving	5	1	2	5	Medication (ibuprofen)	Bilateral back pain with flexion, Rt. side rotation ROM
28/F, 1 month, idiopathic	6	0	3	28	Medication (acetaminophen)	Bilateral back pain with flexion, extension LOM, pain aggravation with sitting, standing
62/F, 3 weeks, idiopathic	4	1	4	15	Medication (ibuprofen), acupuncture	Lt. buttock pain, difficulty sitting
36/M, 2 months, idiopathic	6	0	3	8	Lumbar transforaminal nerve block, osteopathic Tx, medication (acetaminophen)	Lt. side vertical back pain with flexion, extension ROM, Pain aggravation with coughing, standing
70/F, three years, 2 weeks ago sudden aggravation	4	0	2	3	Medication (NSAIDs)	Bilateral back and buttock pain Pain aggravation from sitting to standing (position change)
34/F, 4 months, idiopathic	4	0	6	35	Medication (ibuprofen, dietary supplement), acupuncture	transverse back pain from flexion to extension ROM
51/M, 6 months, after strain	6	0	2	7	Medication (ibuprofen), lumbar steroid root block	Rt. side vertical back pain, aggravation with flexion ROM
52/M, 2 months, gradually	6	0	2	7	Medication (NSAIDs), Acupuncture	Bilateral back pain with Rt. buttock pain Severe in the morning Aggravation from sitting to standing motion
Means	5.588	0.294	3.647	13.529		

VAS, visual analog scale; Tx, treatment; F, female; M, male; NSAIDs, non-steroidal anti-inflammatory drugs; ROM, range of motion; LOM, limitation of range of motion; Lt., left; Rt., right.

#### Exclusion criteria

Patients who underwent surgery or had radiological findings (including MRI) of nerve compression lesions, severe stenotic lesions, vertebral fractures, or infectious lesions requiring surgical intervention were excluded. Patients suspected of myofascial pain syndrome radiologically and clinically caused by other lower back, buttock, and thigh muscles, or patients refusing treatment were also excluded.

### Procedure

After obtaining the history of the lower back and/or buttock pain, we performed physical examinations using standing tests to help narrow the differential diagnosis, including: forward bending (flexion), extension, and both sides lateral side-bending and trunk rotation. Subsequently, the patients were positioned in the lateral decubitus position with the affected or more painful side upward, the upper knee behind the other knee with slight knee flexion, and the affected side arm upward and comfortably stretched over the head. This posture made it easier to palpate the deeply located quadratus lumborum TrPs or lesions by maximally extending the space between the twelfth rib and the iliac crest.

**Figure 1:**
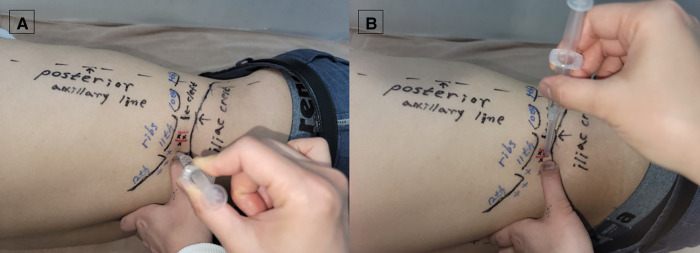
Needle entry point and trajectory for the treatment of (**A**) superficial and (**B**) deep fiber trigger points. (**A**) When we targeted the superficial fibers (iliocostal fibers), palpation was performed slightly obliquely anteriorly (10–20°). To prevent pneumothorax, the needle should not cross the contour of the rib or be inserted in the cranial direction. (**B**) When we targeted the deep fibers (iliolumbar, lumbocostal fibers) near the cleft, we palpated vertically toward the transverse processes of the lumbar vertebrae. If the needle is inserted too anteriorly or at the ventral position instead of in the cleft between the firm or hard back muscle and the soft abdominal muscle, intraperitoneal and retroperitoneal organ injuries can occur; thus, careful needling is required. (The model in Figure 1. is one of the authors of this study.).

Patients were instructed to say “yes” or “ah” if they felt pain when the examiner's thumb pressure was applied to the quadratus lumborum. Those locations were set as target points. Tender points and symptom-related fibrous nodules, taut bands, or lesions were located and labeled with a marking pen. Subsequently, we considered the quadratus lumborum muscle fiber direction and location. When we targeted the superficial fibers (iliocostal fibers), palpation was performed slightly obliquely anteriorly (10–20°). When we targeted the deep fibers (iliolumbar or lumbocostal fibers) near the cleft, we palpated vertically toward the transverse processes of the lumbar vertebrae (Figures 1A,B). We used a newly designed 60–90-mm long, 28G hypodermic needle with a needle guide. Once the needle tip penetrated the TrPs or touched the transverse process, 0.5–1 ml of lidocaine (0.5%) or a mixture of lidocaine (0.5%) and 12.5%–15% dextrose (2% lidocaine, 50% dextrose diluted with normal saline) was injected at each lesion site, including the TrPs, fibrous tissue, and bone attachment site. When the needle reached the target point and visible local twitch responses (LTRs) were elicited and/or the patient experienced pain, we subsequently administered the injectate. Steroids and fluoroscopy or ultrasonography guidance were not used.

Additionally, injection near the L2 and L3 transverse processes and cephalad of the L1 transverse process must be carefully undertaken to avoid kidney injury and pneumothorax by penetrating the diaphragm and pleura, directing the needle tip in a cephalad or cranial direction toward the twelfth rib. Pneumothorax may occur if treated without accurately confirming the twelfth rib.

We did not apply post-isometric muscle relaxation or a moist hot pack over the muscle after injection.

### Newly designed needle and guide tube

**Figure 2:**
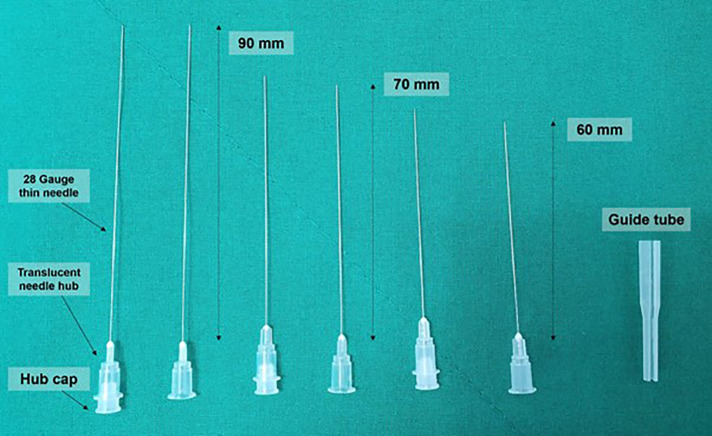
Newly designed 28G 60–90-mm thin hypodermic needles. Newly designed 28G 60–90-mm thin hypodermic needles were used. New needles with a transparent plastic needle hub and hub cap prevented complications, such as unintentional nerve and vessel injuries by the needles or by anesthetic injection, by confirming the presence or absence of blood or cerebrospinal fluid without the need for aspiration. The needle guide is used to adjust the direction of the needle or for direction guidance.

Newly designed 28G 60–90-mm thin hypodermic needles (Figure 2) were utilized for the procedure. New needles with a translucent plastic needle hub and hub cap prevented complications such as unintentional nerve and vascular injuries by the needles or by anesthetic injection by confirming the existence or non-existence of cerebrospinal fluid or blood without the need for aspiration.

The very thin 28G 60–90-mm needle with or without a hub cap would bend or could not penetrate the skin during the insertion process without a guide tube. Therefore, the guide tube was used to adjust the direction of the needle or provide directional guidance and then pulled sideways and removed when the upper part of the injection needle shaft remained 0.5–1 cm from the top of the guide tube.

A patent for the newly devised needle (injection needle with a plastic needle hub cap) and guide tube has been registered with the Korean Intellectual Property Office; inventor, author, Hun Man Sirh. A patent for the guide tube (puncturing guidance tube) has also been registered with the United States Patent and Trademark Office.

### Five landmarks for effective and safe procedures

**Figure 3:**
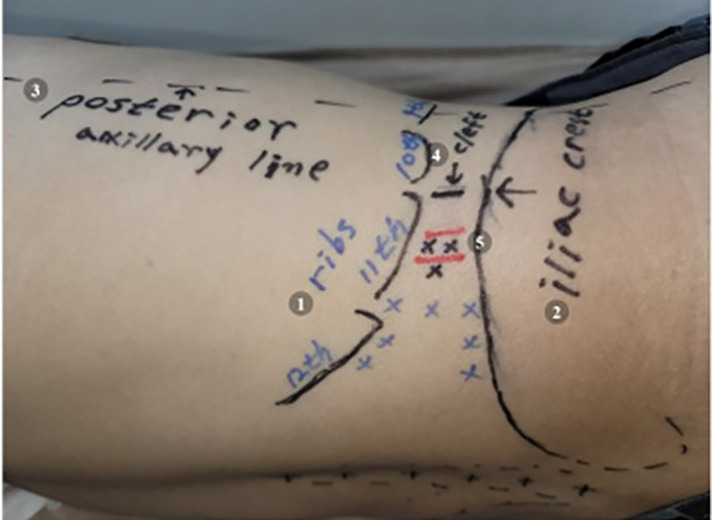
Five landmarks for an effective and safe procedure. The most important aspect of an effective and safe procedure is to check the five landmarks accurately. (1) the contour of the tip of the twelfth rib and the lower margin, (2) the iliac crest, (3) the posterior axillary line, (4) the cleft between the firm or hard back muscle and the soft abdominal muscle, (5) the lumbar transverse process (located 1 cm–1.5 cm behind the cleft) in thin patients. Black x marks denote the needle entry point when targeting deep fiber TrPs, blue x marks denote the needle entry point when targeting superficial fiber TrPs, and red lines denote the lumbar transverse process, which is generally located 1 cm–1.5 cm posterior to the cleft described in (4). (The model in Figure 3. is one of the authors of this study.).

We used five easily identifiable and important landmarks (Figure 3) to treat the quadratus lumborum muscle lesions effectively and safely and to avoid pneumothorax (above the first lumbar transverse process) and intra-abdominal organ injury (above the second or third lumbar transverse process). The most important factors are locating the contour of the tip of the twelfth rib and its lower margin, the iliac crest, the posterior axillary line, and the cleft between the firm or hard back muscle and the soft abdominal muscle, and possibly, to palpate the lumbar transverse process (located 1 cm–1.5 cm behind the cleft) in thin patients. The tip of the twelfth rib does not extend beyond the posterior axillary line. Therefore, it is important to find the tip of the twelfth rib behind the posterior axillary line. The cleft between the firm back muscle and the soft abdominal muscle is an important landmark to avoid abdominal visceral injury by the needle. The lumbar transverse processes are located 1 cm–1.5 cm behind the cleft and do not exist in front of (anterior to) the cleft. The midpoint between the twelfth rib and the iliac crest usually corresponds to the third lumbar vertebral level (L3), and the risk of kidney damage is low because the kidneys do not generally descend below the L3 level, even if there is a variation in the kidney location.

### Assessment

We retrospectively reviewed the electronic medical records from Dr. Sirh's Pain Clinic. We evaluated patients' self-reported pain intensity scores using a 0–10 point visual analog scale (VAS) score before and after the procedure, visible and invisible LTRs, and patients' pain report during the procedure as outcome measures. Treatments were administered one to three times per week for the first few treatments, depending on the severity of the symptoms and lesion complexity (TrP size, number, and location), and were subsequently tapered to once a week and once every two to three weeks as symptoms improved. We evaluated the number of treatments until the end of the procedure. Data in this brief report were analyzed descriptively due to small sample size.

## Results

Patients were on average 47.35 years (range: 28–70); 11 were men and 6 were women. The duration of symptom onset ranged from three days to three years. The mean pain duration was 163.2 ± 296.6 days. Among the 17 patients, 10 had bilateral lesions and 7 had unilateral lesions. Patients were not treated bilaterally at each treatment session.

Acute and subacute patients received an average of 3.6 (range 2–4) procedures; Chronic patients received an average of 4.9 (range 2–8). Treatments were repeated according to the severity and duration of the symptoms.

All patients reported almost complete resolution of lumbar and/or buttock pain (Table 1) and/or paresthesia after treatment. The VAS score decreased from 4 to 8/10 (mean score: 5.588) points to 0–1/10 point at the end of treatment (mean score: 0.294) with improvement in range of motion. All patients reported discontinuing analgesic drugs. Other than injection-related pain and negligible bleeding or bruising, which was rare, minor, and self-limited, we observed no complications or side effects.

## Discussion

This brief research report describes the importance of the quadratus lumborum muscle TrPs and enthesopathy in nonspecific lower back and/or buttock pain and also describes a palpation-guided approach technique using five landmarks and its advantages for safely and effectively performing TPI and prolotherapy. This study suggests that landmark-based blind TPIs may be used for patients with intractable lumbar pain, buttock pain, and/or paresthesia due to quadratus lumborum muscle TrPs, fibrosis, calcification, and tears or other tendon lesions.

We determined the diagnosis using six screening standing examinations, and treatment effectiveness using before-and-after treatment assessment of clinical symptoms and signs, loss of referred pain, improvement of the limited range of motion, and degree of symptom relief (decrease of VAS score). In almost all patients, tender spots in the quadratus lumborum muscle were identified prior to treatment. Injection was performed at tender points and/or palpable taut bands, and an LTR and immediate symptomatic improvement (limited range of motion and referred pain) were almost always confirmed during needling and immediately after treatment. In our study, most quadratus lumborum muscle lesions were tender and diagnosed as myofascial TrPs and enthesopathy or fibrosis with a tight or firm sensation, resulting in increased resistance to needling. Therefore, during injection, the needle tips must be in contact with the transverse process or iliac crest for the treatment of enthesopathy.

When clinical history and physical examination are considered, myofascial lower back pain and patterns of quadratus lumborum TrPs are characterized by pain on coughing or sneezing with upper iliocostal fiber TrPs, vertical pain with a unilateral lesion and/or transverse pain with a bilateral lesion, and a limitation of motion, such as forward bending (mainly), turning, leaning to the opposite side, rolling onto the side from the supine position, climbing stairs, arising from the supine position, or standing up from a chair (10). Quadratus lumborum myofascial pain syndrome generally does not produce radiating pain and/or paresthesia to the leg. However, when combined with sacroiliac joint lesions or gluteus minimus TrPs, it mimics disc radiculopathy. The associated myofascial TrPs of the quadratus lumborum may develop secondarily in other muscles, such as the contralateral quadratus lumborum, ipsilateral iliopsoas, iliocostalis between T11 and L3, or as satellite TrPs (gluteus medius and minimus muscles) in its pain reference zones. Furthermore, quadratus lumborum TrPs may be related to articular dysfunction (10). Therefore, lower back muscles, particularly the quadratus lumborum muscle, should always be included in differential diagnosis for the effective treatment of lower back pain and/or buttock pain.

Pain due to myofascial pain syndrome and enthesopathy has received less attention than other lower back and/or buttock pain diagnoses regardless of thigh pain due to the lack of an appropriate and accurate diagnosis. Diagnosis of erector spinae and quadratus lumborum muscle lesions is vitally important in lower back pain. Considering the aforementioned characteristic pain and patterns of quadratus lumborum TrPs, a differential diagnosis considering the quadratus lumborum muscle and other back muscles, such as the erector spinae and iliopsoas, is required to accurately evaluate and treat quadratus lumborum lesions in back and/or buttock pain.

Some reports (15, 16) have recommended ultrasound-guided TPI considering its advantages, such as high TrP detection rate, reliability, diagnostic objectivity, and reduction of complications. This is because the physical diagnosis of conventional blind TPI for myofascial pain syndrome has traditionally depended on the physician's ability to identify myofascial TrPs (16). However, this study suggests that, when its advantages, efficacy, and frequency of complications are considered, landmark-based blind injection may not be inferior to ultrasound-guided TPI.

Furthermore, in essence, the ultrasound-guided quadratus lumborum injection technique for pain management targets interfascial plane blockage or hydrodissection by identifying markers of muscle or bony landmarks, whereas quadratus lumborum myofascial pain syndrome primarily involves TrPs, fibrosis, calcification, and enthesopathy. These are also difficult to distinguish from the surrounding lesions by ultrasound. Lower back muscles, including the quadratus lumborum, are usually bulky and deeply seated. Therefore, to address the aforementioned lesions, treatment through physical examination and delicate palpation may be more important. Delicate palpation and anatomical knowledge based on five landmarks enable physicians to treat lower back myofascial pain syndrome easily, safely, and effectively with or without ultrasonography or fluoroscopy because quadratus lumborum TrPs cannot usually be visualized with image guidance. We have always confirmed and evaluated LTRs or patient reports of pain (jump signs) when inserting the needle into TrPs or taut bands.

Although ultrasonography is known to be more helpful in identifying LTRs and guiding injections in deeper muscles (16), it must be used primarily to search for symptom-related lesions, such as TrPs or taut bands, including tender or non-tender bands under manual palpation. It is also required to confirm visible and invisible (slight muscle twitch felt by applying deep pressure with the fingertip) LTRs during needling into TrPs (15, 16) under landmark or ultrasound guidance. As such, palpation for TrP diagnosis remains a key process, and at least two or three of the criteria, such as a tender taut band, a hypersensitive spot, referred pain, and LTRs, are required for TrP diagnosis (17, 18). To effectively treat myofascial pain syndrome, we inserted the needle toward the tender points in the same trajectory as we palpated and then confirmed visible and invisible LTRs or a strong pain response reported by the patient (jump sign) (Supplementary Videos 1, 2).

It should not be overlooked that landmark-based intramuscular TPI through a lateral approach could significantly improve quadratus lumborum myofascial pain. It can be performed easily even at the bedside in the emergency room or ward where ultrasound equipment is not available. For ligament and tendon lesions, bony contact of the needle with the lumbar transverse processes or iliac crest is required. Travell & Simons (10) described the quadratus lumborum TPI without a concrete approach method or landmarks, which are necessary to perform needling safely and effectively. Therefore, quadratus lumborum myofascial pain syndrome has been largely overlooked and has not been treated satisfactorily, either blind or with image guidance using ultrasound and fluoroscopy.

Despite their advantages, MRI and ultrasound also have shortcomings, such as revealing findings unrelated to pain or symptoms. Furthermore, in ultrasound-guided TPI, it is difficult to accurately approach palpated tender points, and high-resolution devices and long-term learning curves are needed to improve treatment quality. In the future, additional studies are required to prospectively compare our palpation-guided, landmark-based integrated injection method with ultrasound-guided injection for low back pain, including quadratus lumborum myofascial pain syndrome.

Additionally, we did not observe significant complications or side effects, such as infection or lumbar plexus and organ injury, except for negligible bleeding and bruising, because of the use of the newly devised 60–90-mm 28G needle and guide tube, and our approach method using five landmarks. However, potential side effects include infection, bruising, pneumothorax, lumbar plexus injury, hypotension, and nausea due to unintentional sympathetic ganglion block. Therefore, it is safer to treat the quadratus lumborum unilaterally in a single treatment session.

This study had certain limitations. First, this study did not include an adequate number of patients for statistical comparative analysis. Second, the data were retrospectively reviewed. Third, as most patients with quadratus lumborum muscle problems typically also exhibit myofascial pain in other lower back muscles, lumbar facet joint problems, and hip joint problems, we did not enroll patients who were simultaneously receiving treatment for these issues. Fourth, our study is single-arm without a control group and did not assess differences between acute, subacute, and chronic cases. Fifth, the other limitation of our study was that it lacked long-term follow-up. A larger, controlled, prospective study is needed in the future to evaluate the effects of our TPI and integrated injection technique in patients with combined problems of various structures and the quadratus lumborum.

In conclusion, myofascial pain and enthesopathy of the quadratus lumborum muscle are considerably more important as causes of lower back and/or buttock pain than is commonly realized and should be differentially diagnosed. Delicate palpation with proper pressure, position, and needling technique using the above-mentioned five landmarks are important for the diagnosis and treatment of quadratus lumborum muscle lesions. This study suggests that a safer and more effective treatment can be provided through a repeated and integrated injection method using palpation-guided technique based on five landmarks with the newly designed needle and guide tube, even without image guidance.

## Data Availability

The original contributions presented in the study are included in the article/**Supplementary Material**, further inquiries can be directed to the corresponding author/s.

## References

[1] GBD 2017 Disease and Injury Incidence and Prevalence Collaborators. Global, regional, and national incidence, prevalence, and years lived with disability for 354 diseases and injuries for 195 countries and territories, 1990–2017: a systematic analysis for the global burden of disease study 2017. Lancet. (2018) 392:1789–858. 10.1016/S0140-6736(18)32279-7 30496104 PMC6227754

[2] Airaksinen O Brox JI Cedraschi C Hildebrandt J Klaber-Moffett J Kovacs F Chapter 4. European guidelines for the management of chronic nonspecific low back pain. Eur Spine J. (2006) 15(Suppl 2):S192–300. 10.1007/s00586-006-1072-1 16550448 PMC3454542

[3] Balagué F Mannion AF Pellisé F Cedraschi C. Non-specific low back pain. Lancet. (2012) 379:482–91. 10.1016/S0140-6736(11)60610-7 21982256

[4] Becker A Held H Redaelli M Strauch K Chenot JF Leonhardt C Low back pain in primary care: costs of care and prediction of future health care utilization. Spine (Phila Pa 1976). (2010) 35:1714–20. 10.1097/BRS.0b013e3181cd656f 21374895

[5] Deyo RA Weinstein JN. Low back pain. N Engl J Med. (2001) 344:363–70. 10.1056/NEJM200102013440508 11172169

[6] Matsumoto J Isu T Kim K Iwamoto N Morimoto D Isobe M. Surgical treatment of middle cluneal nerve entrapment neuropathy: technical note. J Neurosurg Spine. (2018) 29:208–13. 10.3171/2017.12.SPINE17991 29775161

[7] Vagaska E Litavcova A Srotova I Vlckova E Kerkovsky M Jarkovsky J Do lumbar magnetic resonance imaging changes predict neuropathic pain in patients with chronic non-specific low back pain? Medicine (Baltimore). (2019) 98:e15377. 10.1097/MD.0000000000015377 31027128 PMC6831323

[8] Teixeira MJ Yeng LT Garcia OG Fonoff ET Paiva WS Araujo JO. Failed back surgery pain syndrome: therapeutic approach descriptive study in 56 patients. Rev Assoc Med Bras (1992). (2011) 57:282–7. 10.1016/S0104-4230(11)70060-4 21691691

[9] Phillips S Mercer S Bogduk N. Anatomy and biomechanics of quadratus lumborum. Proc Inst Mech Eng H. (2008) 222:151–9. 10.1243/09544119JEIM266 18441751

[10] Travell JG Simons DG. Myofascial pain and dysfunction: The trigger point manual. Baltimore: Williams & Wilkins (1999). 28–88. 168–85.

[11] Hackett GS Hemwall G Montgomery G. Ligament and tendon relaxation. Wisconsin: Hackett Hemwall Foundation (2008). 3–62.

[12] Akerman M Pejčić N Veličković I. A review of the quadratus lumborum block and ERAS. Front Med (Lausanne). (2018) 5:44. 10.3389/fmed.2018.00044 29536008 PMC5834926

[13] Rabago D Reeves KD Doherty MP Fleck M. Prolotherapy for musculoskeletal pain and disability in low- and middle-income countries. Phys Med Rehabil Clin N Am. (2019) 30(4):775–86. 10.1016/j.pmr.2019.07.003 31563169

[14] Sirh SJ Sirh SW Mun HY Sirh HM. Integrative treatment for tinnitus combining repeated facial and auriculotemporal nerve blocks with stimulation of auditory and non-auditory nerves. Front Neurosci. (2022) 16:758575. 10.3389/fnins.2022.758575 35299621 PMC8923298

[15] Kumbhare D Singh D Rathbone HA Gunn M Grosman-Rimon L Vadasz B Ultrasound-guided interventional procedures: myofascial trigger points with structured literature review. Reg Anesth Pain Med. (2017) 42:407–12. 10.1097/AAP.0000000000000572 28277418

[16] Rha DW Shin JC Kim YK Jung JH Kim YU Lee SC. Detecting local twitch responses of myofascial trigger points in the lower-back muscles using ultrasonography. Arch Phys Med Rehabil. (2011) 92:1576–80.e1. 10.1016/j.apmr.2011.05.005 21839982

[17] Fernández-de-Las-Peñas C Dommerholt J. International consensus on diagnostic criteria and clinical considerations of myofascial trigger points: a delphi study. Pain Med. (2018) 19:142–50. 10.1093/pm/pnx207 29025044

[18] Simons DG. Diagnostic criteria of myofascial pain caused by trigger points. J Musculoskelet Pain. (1999) 7:111–20. 10.1300/J094v07n01_11

